# Mortality and Outcomes in Cerebrovascular Disease Patients With Emphasis on COVID-19: A Cross-Sectional Analysis of the National Inpatient Sample 2020

**DOI:** 10.7759/cureus.42806

**Published:** 2023-08-01

**Authors:** Adishwar Rao, Akriti Agrawal, Trisha Chatterjee

**Affiliations:** 1 Department of Epidemiology, Human Genetics and Environmental Sciences, The University of Texas Health Science Center at Houston School of Public Health, Houston, USA; 2 Department of General Medicine, Netaji Subhash Chandra Bose Medical College, Jabalpur, IND

**Keywords:** united states of america, national inpatient sample database, sars-cov-2, mortality, cerebrovascular disease, covid-19

## Abstract

Background

COVID-19-related pulmonary complications have been explored extensively in the recent past. There is also a significant amount of literature on the neurological manifestations of COVID-19. However, there exists an unmet need to assess the impact of COVID-19 on patients with cerebrovascular diseases and its role in affecting mortality in such patients.

Methods

In this cross-sectional study, we analyzed 401,318 hospitalized patients with cerebrovascular diseases using the discharge data from the National Inpatient Sample 2020 to assess the association of COVID-19 with multiple clinical conditions, along with additional factors, such as length of stay in the hospital, total charges incurred, region and type of hospital, and primary insurance/payer in the United States of America. We used a multivariable logistic regression model to predict factors relating to mortality in such patients.

Results

The mortality during hospitalization in patients with cerebrovascular disease who were also diagnosed with COVID-19 was significantly higher than the patients without COVID-19 (22.50% vs 5.44%, p-value <0.0001). COVID-19 independently increased the odds of death significantly in patients with cerebrovascular diseases (adjusted OR = 4.81, p-value <0.0001). Other statistically and clinically significant factors that contributed to increased odds of mortality in such patients were comorbidities such as moderate/severe liver disease, myocardial infarction, congestive heart failure, and complications such as the development of a saddle pulmonary embolus.

Conclusion

COVID-19 was associated with higher mortality in patients with cerebrovascular diseases. It also significantly increased the duration of hospital stay and odds of mortality in such patients.

## Introduction

COVID-19 is a catastrophic viral illness caused by SARS-CoV-2 that originated in Wuhan, China, and took the entire global ecosystem by storm in 2019 and 2020 [1]. It rapidly transformed into a pandemic, leaving lasting impacts on the global healthcare industry and economy [2]. The United States has been one of the most affected countries in the world by COVID-19-related morbidity and mortality. Despite the development of multiple vaccines and therapeutic modalities for COVID-19, the damage incurred may be irreversible. To prevent similar pandemics from sprouting in the future, it is imperative to analyze and assess the factors that contributed to significant mortality in this pandemic. SARS-CoV-2 is a single-stranded, positive-sense, enveloped RNA virus [3-4] that primarily interferes with the functioning of the angiotensin-converting enzyme 2 (ACE2). It causes a predominantly respiratory illness but may also lead to multiorgan dysfunction, systemic inflammation, cytokine storm, hypercoagulability, and death [1]. While many infections are asymptomatic [5], one of the most clinically apparent presentations of severe COVID-19 is COVID-19-associated pneumonia, particularly in patients with altered immune function and/or pre-existing comorbidities [3]. The major non-pulmonary manifestations may involve the cardiovascular, gastrointestinal, hepatobiliary, renal, or neurological systems [6]. Although existing studies have explored the development of acute cerebrovascular and neurological complications in patients with COVID-19 [7], there is a dearth of studies on the impact of COVID-19 in patients with pre-existing cerebrovascular disease. Therefore, in this study, we assess the impact of COVID-19 along with certain relevant comorbidities, such as myocardial infarction, congestive heart failure, moderate/severe liver disease, and complications, such as the development of a saddle pulmonary embolus during the duration of hospital stay, on mortality in patients with pre-existing or concomitantly diagnosed cerebrovascular disease. We also explored the association of death in patients with cerebrovascular disease who also suffered from COVID-19, with duration of stay at the hospital, age at the time of hospitalization, primary insurance/payer, hospital type (region and location), race and gender, among other relevant factors.

## Materials and methods

The data were sourced from the publicly available discharge data of the National Inpatient Sample 2020, obtained from the Healthcare Cost and Utilization Project (HCUP), Agency for Healthcare Research and Quality [8]. A cohort of patients with either a primary or any of the secondary diagnoses of cerebrovascular disease was selected using the relevant International Classification of Diseases, Tenth Revision, Clinical Modification (ICD-10-CM) codes (Appendix 1) [9]. We conducted a cross-sectional study and included patients diagnosed with cerebrovascular disease aged ≥15 years at the time of hospital admission. The cohort was then classified based on a diagnosis of COVID-19 [9]. The patients were also divided into three age categories, 15 to less than 45 years, 45 to less than 65 years, and greater than or equal to 65 years. The Charlson Comorbidity Index (CCI) was used to assess the impact of comorbidities on mortality in patients with cerebrovascular disease, and a CCI greater than four was considered to be clinically significant. The categorical variables were analyzed using the Pearson Chi-square test to determine the baseline characteristics of the patients and assess associations with COVID-19. A multivariable logistic regression model was used to determine predictors of mortality in patients with cerebrovascular disease, focusing on conditions like COVID-19, moderate/severe liver disease, presence of a saddle pulmonary embolus, myocardial infarction, and congestive heart failure. Stata/BE 17.0 for Mac was used for this statistical analysis [10]. Statistical significance was set at 0.05. Approval from Institutional Review Boards (IRB) was not required due to the nature of the publicly available database provided by HCUP [11].

## Results

A total of 401,318 hospitalized patients in the US in 2020 with a primary or secondary diagnosis of cerebrovascular disease were included in this study. A total of 17,440 (4.35%) patients were also diagnosed with COVID-19, while 383,878 (95.65%) were not. Amongst the patients who had COVID-19, 612 (3.51%) were 15 to <45 years old, 4,445 (25.49%) were 45 to <65 years old, and 12,383 (71%) were ≥65 years old. While amongst those without COVID-19, 19,682 (5.13%) were 15 to <45 years old, 106,296 (27.69%) were 45 to <65 years old, and 257,900 (67.18%) were ≥65 years old. A total of 7,720 (44.27%) patients who had COVID-19 were females, while 187,349 (48.81%) patients who did not have COVID-19 were females. Amongst the patients with COVID-19, 8,545 (50.46%) were racially or ethnically White, 4,449 (26.27%) were Black, 2,506 (14.80%) were Hispanic, 658 (3.89%) were Asian or Pacific Islander, 116 (0.69%) were Native American, and 659 (3.89%) were identified as Other. In contrast, amongst those without COVID-19, 250,054 (66.84%) were racially or ethnically White, 68,850 (18.40%) were Black, 31,240 (8.35%) were Hispanic, 11,519 (3.08%) were Asian or Pacific Islander, 2,024 (0.54%) were Native American, and 10,407 (2.78%) were identified as Other. Amongst the COVID-19 patient group, 12,312 (70.73%) used Medicare, 1,950 (11.20%) used Medicaid, 2,283 (13.12%) used Private Insurance, 332 (1.91%) paid themselves, 23 (0.13%) were not charged, and 506 (2.91%) used some other primary payer. From the patient group without COVID-19, 259,741 (67.75%) used Medicare, 40,059 (10.45%) used Medicaid, 62,124 (16.20%) used Private Insurance, 11,069 (2.89%) paid themselves, 872 (0.23%) were not charged, and 9,537 (2.49%) used some other primary payer. Hospitals in the Northeast region of the US catered to 3,510 (20.13%) patients with COVID-19 and 66,660 (17.36%) patients without COVID-19. Those in the Midwest catered to 3,946 (22.63%) COVID-19 patients and 84,953 (22.13%) patients without COVID-19. Those in the South catered to 7,016 (40.23%) COVID-19 patients and 160,309 (41.76%) patients without COVID-19. While those in the West catered to 2,968 (17.02%) COVID-19 patients and 71,956 (18.74%) patients without COVID-19. Amongst the patients with COVID-19, 1,380 (7.91%) were admitted to a rural hospital, 2,731 (15.66%) to an urban nonteaching hospital, and 13,329 (76.43%) to an urban teaching hospital. In contrast, amongst those without COVID-19, 27,645 (7.20%) were admitted to a rural hospital, 63,072 (16.43%) to an urban nonteaching hospital, and 293,161 (76.37%) to an urban teaching hospital. A total of 9,552 (54.77%) patients with COVID-19 stayed for seven or more days at the hospital, and 7,888 (45.23%) for less than seven days, while 121,052 (31.53%) patients without COVID-19 stayed for seven or more days at the hospital and 262,826 (68.47%) for less than seven days. Amongst the patients with COVID-19, 6,826 (39.14%) incurred charges worth <$50,000, 4,335 (24.86%) incurred between ≥$50,000 and <$100,000, and 6,279 (36%) incurred ≥$100,000. Amongst those who did not have COVID-19, 184,024 (47.94%) incurred charges worth <$50,000, 100,293 (26.13%) incurred between ≥$50,000 and <$100,000, and 99,561 (25.94%) incurred ≥$100,000. Out of the COVID-19 patients, 2,383 (13.66%) had a myocardial infarction, 4,771 (27.36%) had congestive heart failure, 131 (0.75%) had moderate/severe liver disease, and 21 (0.12%) had a saddle pulmonary embolus. While in those without COVID-19, 53,911 (14.04%) had a myocardial infarction, 101,801 (26.52%) had congestive heart failure, 3,350 (0.87%) had moderate/severe liver disease, and 351 (0.09%) had a saddle pulmonary embolus. A total of 8,528 (48.90%) COVID-19 patients had a CCI ≥4, while 183,723 (47.86%) patients without COVID-19 had CCI ≥4. A total of 3,920 (22.50%) patients with cerebrovascular disease who had COVID-19 died, while 20,873 (5.44%) patients with cerebrovascular disease who did not have COVID-19 died. COVID-19 was statistically significantly associated with age group (p<0.0001), gender (p<0.0001), race (p<0.0001), primary payer (p<0.0001), region of hospital (p<0.0001), location and teaching status of hospital (p<0.0001), length of stay (grouped) (p<0.0001), total charges incurred (grouped) (p<0.0001), congestive heart failure (p = 0.014), CCI (p = 0.007), and mortality during hospitalization (p<0.0001). These results are summarized in Table 1.

**Table 1:** Key summary statistics for the study cohort of patients with cerebrovascular diseases and comparison between COVID-19 and non-COVID-19 groups. ^a^Pearson Chi-Square test ^b^Charlson Comorbidity Index

Characteristic	Study cohort (n = 401,318)		P-value^a^
	COVID-19 cohort	Non-COVID-19 cohort	
n (%)	17,440 (4.35)	383,878 (95.65)	
Age group, n (%)			<0.0001
15 to <45 years	612 (3.51)	19,682 (5.13)	
45 to <65 years	4,445 (25.49)	106,296 (27.69)	
≥65 years	12,383 (71.00)	257,900 (67.18)	
Female, n (%)	7,720 (44.27)	187,349 (48.81)	<0.0001
Race/Ethnicity, n (%)			<0.0001
White	8,545 (50.46)	250,054 (66.84)	
Black	4,449 (26.27)	68,850 (18.40)	
Hispanic	2,506 (14.80)	31,240 (8.35)	
Asian or Pacific Islander	658 (3.89)	11,519 (3.08)	
Native American	116 (0.69)	2,024 (0.54)	
Other	659 (3.89)	10,407 (2.78)	
Primary Payer (Insurance), n (%)			<0.0001
Medicare	12,312 (70.73)	259,741 (67.75)	
Medicaid	1,950 (11.20)	40,059 (10.45)	
Private Insurance	2,283 (13.12)	62,124 (16.20)	
Self-pay	332 (1.91)	11,069 (2.89)	
No charge	23 (0.13)	872 (0.23)	
Other	506 (2.91)	9,537 (2.49)	
Region of hospital, n (%)			<0.0001
Northeast	3,510 (20.13)	66,660 (17.36)	
Midwest	3,946 (22.63)	84,953 (22.13)	
South	7,016 (40.23)	160,309 (41.76)	
West	2,968 (17.02)	71,956 (18.74)	
Location and teaching status of hospital, n (%)			<0.0001
Rural	1,380 (7.91)	27,645 (7.20)	
Urban non-teaching	2,731 (15.66)	63,072 (16.43)	
Urban teaching	13,329 (76.43)	293,161 (76.37)	
Length of stay - grouped, n (%)			<0.0001
≥7 days	9,552 (54.77)	121,052 (31.53)	
<7 days	7,888 (45.23)	262, 826 (68.47)	
Major Complications/Comorbidities, n (%)			
Myocardial Infarction	2,383 (13.66)	53,911 (14.04)	0.158
Congestive Heart Failure	4,771 (27.36)	101,801 (26.52)	0.014
Moderate/Severe Liver Disease	131 (0.75)	3,350 (0.87)	0.091
Saddle Pulmonary Embolus	21 (0.12)	351 (0.09)	0.219
CCI^b^ ≥4	8,528 (48.90)	183,723 (47.86)	0.007
Mortality during hospitalization	3,920 (22.50)	20,873 (5.44)	<0.0001

The results of the multivariable logistic regression suggested that patients with cerebrovascular disease who also had COVID-19 had significantly higher odds of mortality than patients without COVID-19 (adjusted OR = 4.81, p<0.0001). Similar clinically important results were seen for several groups: those with a length of hospital stay ≥7 days (adjusted OR = 1.38, p<0.0001); patients aged ≥65 years (adjusted OR = 1.20, p<0.0001); and patients who identified as Hispanic (adjusted OR = 1.10, p<0.0001), Asian or Pacific Islander (adjusted OR = 1.36, p<0.0001), or Other (adjusted OR = 1.32, p<0.0001). The same trends held for patients with moderate/severe liver disease (adjusted OR = 3.03, p<0.0001), saddle pulmonary embolus (adjusted OR = 2.80, p<0.0001), myocardial infarction (adjusted OR = 1.38, p<0.0001), congestive heart failure (adjusted OR = 1.27, p<0.0001), or a CCI ≥4 (adjusted OR = 1.18, p<0.0001). Increased odds of death were statistically significant with all primary payers, with patients who used Medicare as the reference group. These results are summarized in Table 2.

**Table 2:** Results of a multivariate logistic regression model predicting mortality in cerebrovascular disease patients in 2020. ^a^Odds Ratio (± Standard Error) ^b^95% Confidence Interval ^c^β-coefficient for the respective predictors ^d^Charlson Comorbidity Index

Predictors	Adjusted OR (±SE)^a^	95% CI^b^	β^c^	P-value
COVID-19	4.81 (0.10)	(4.62, 5.01)	1.57	<0.0001
Length of hospital stay, ≥7 days	1.38 (0.02)	(1.34, 1.42)	0.32	<0.0001
Age group				
15 to <45 years	Reference			
45 to <65 years	0.81 (0.28)	(0.76, 0.87)	-0.21	<0.0001
≥ 65 years	1.20 (0.04)	(1.12, 1.28)	0.18	<0.0001
Gender, Female	0.99 (0.01)	(0.96, 1.02)	-0.01	0.427
Race/Ethnicity				
White	Reference			
Black	0.90 (0.02)	(0.87, 0.94)	-0.10	<0.0001
Hispanic	1.10 (0.03)	(1.05, 1.15)	0.09	<0.0001
Asian or Pacific Islander	1.36 (0.05)	(1.27, 1.46)	0.31	<0.0001
Native American	1.18 (0.10)	(1.00, 1.39)	0.16	0.055
Other	1.32 (0.05)	(1.23, 1.42)	0.28	<0.0001
Primary Payer (Insurance)				
Medicare	Reference			
Medicaid	1.26 (0.04)	(1.20, 1.33)	0.23	<0.0001
Private Insurance	1.32 (0.03)	(1.26, 1.37)	0.27	<0.0001
Self-pay	1.84 (0.07)	(1.70, 1.99)	0.61	<0.0001
No charge	1.41 (0.21)	(1.05, 1.88)	0.34	0.020
Other	2.13 (0.08)	(1.99, 2.28)	0.76	<0.0001
Comorbidities and Complications				
Myocardial Infarction	1.38 (0.02)	(1.33, 1.43)	0.32	<0.0001
Congestive Heart Failure	1.27 (0.02)	(1.23, 1.31)	0.24	<0.0001
Moderate/Severe Liver Disease	3.03 (0.15)	(2.75, 3.32)	1.11	<0.0001
Saddle Pulmonary Embolus	2.80 (0.42)	(2.10, 3.76)	1.03	<0.0001
CCI^d^ ≥4	1.18 (0.02)	(1.15, 1.22)	0.17	<0.0001

## Discussion

Cerebrovascular diseases may commonly present in the setting of a coagulopathic environment [12]. COVID-19 also promotes the development of a severe coagulopathic state, which may exacerbate coagulopathic tendencies in patients with pre-existing cerebrovascular disease [13]. Additionally, SARS-CoV-2 may invade the brain parenchyma and cause depletion of ACE2, which is considered to be protective against neuronal ischemic damage [14]. The combination of enhanced coagulopathic tendencies and decreased neuroprotection may play a vital role in the development and/or progression of cerebrovascular diseases in patients with COVID-19 [15]. Our study analyzed the prevalence, complications, and mortality related to COVID-19 in 401,318 hospitalized patients with a primary or secondary diagnosis of cerebrovascular disease using the National Inpatient Sample 2020 database from HCUP. The most clinically relevant outcomes from our study included a higher proportion of deaths and longer duration of hospital stay in patients with COVID-19, and higher odds of death in the presence of a concomitant diagnosis of COVID-19, presence of comorbidities, such as myocardial infarction, moderate/severe liver disease, congestive heart failure, or complications, such as the development of a saddle pulmonary embolus. The proportion of patients with both cerebrovascular disease and COVID-19 that died was greater than four times than those with cerebrovascular disease without COVID-19 (22.5% vs 5.44%, respectively, p-value<0.0001). A COVID-19 diagnosis in patients with the cerebrovascular disease was also associated with a much greater proportion of patients requiring seven or more days of hospital stay (54.77% vs 31.53%, p-value <0.0001). The proportion of patients who were charged ≥$100,000 was significantly higher in the COVID-19 group (36% vs 25.94%, p-value <0.0001). COVID-19 independently more than quadrupled the odds of death in patients with cerebrovascular disease (adjusted OR = 4.81; p-value <0.0001), while the diagnosis of a saddle pulmonary embolus more than doubled the odds (adjusted OR = 2.80; p-value <0.0001), and moderate/severe liver disease nearly tripled the odds (adjusted OR = 3.03; p-value <0.0001) of death in patients with cerebrovascular disease. Myocardial infarction independently increased the odds of death by 38% (adjusted OR = 1.38; p-value <0.0001), while congestive heart failure increased the odds by 27% (adjusted OR = 1.27; p-value <0.0001). A CCI of four or more increased the odds of death by 18% (adjusted OR = 1.18; p-value <0.0001). Previous studies have suggested that younger patients have a lesser probability of having a cerebrovascular disease, such as stroke, as a complication of COVID-19, compared to older patients [13]. Per our data, older patients, age ≥65 years, independently increased the odds of death by 20% (adjusted OR = 1.20; p-value <0.0001). Amongst the different races, being Asian or Pacific Islander increased the odds of death during hospitalization by 36% (adjusted OR = 1.36; p-value <0.0001). Interestingly, the odds of death were less in the patients aged 45 to <65 years (adjusted OR = 0.81; p-value <0.0001) compared to patients aged 15 to <45 years. Lastly, the patients who opted to pay their hospitalization costs themselves had an 84% increase, the highest increase amongst all forms of primary payers, in odds of death (adjusted OR = 1.84; p-value <0.0001). Additionally, despite the clinically and statistically significant findings, this study, like any other study, is not immune to shortcomings. There are two major limitations to the scope of the application of the results of this study. Firstly, due to the study design, the temporality of the associations cannot be assessed. Secondly, due to the database being an administrative billing database, there is limited clinical insight, with no significant information on follow-ups, drug therapy, laboratory parameters, and imaging. Additionally, a study from three large European countries suggested that the transient reallocation of neurologists towards COVID-19 care units may have played a role in a lower standard of care for stroke patients [16]. It is imperative to explore whether this phenomenon could have also played a role in COVID-19-related mortality in patients with cerebrovascular diseases in the United States. Therefore, the authors of this scientific study urge the conduct of further longitudinal studies to ascertain the temporality of these associations, address these limitations, and account for potential non-clinical confounders.

## Conclusions

The COVID-19 pandemic altered the course of diagnosis and management of patients with cerebrovascular diseases and affected such patients in various ways. On the one hand, it created a favorable environment for up-regulated coagulopathic pathways leading to accelerated thrombosis and coagulation. On the other hand, it collapsed the global medical ecosystem and increased inaccessibility to the adequate standard of care for such patients. Our study principally included hospitalized patients diagnosed with any cerebrovascular disease in the United States, according to the ICD-10-CM coding algorithm. We analyzed the data and explored associations between COVID-19 and other clinical and non-clinical factors. The results obtained suggested that COVID-19 was significantly associated with higher mortality. COVID-19 also emerged as a clinically and statistically significant predictor of mortality that independently increased the odds of death in hospitalized patients with cerebrovascular diseases.

## Appendices

Appendix 1 

**Table 3:** Supplementary table demonstrating the ICD-10-CM codes used. ICD-10-CM: International Classification of Diseases, Tenth Revision, Clinical Modification.

Diagnosis	ICD-10-CM code(s)
Cerebrovascular Diseases	G45, G46, H34.0, I60-I69
COVID-19	U07.1
Myocardial Infarction	I21, I22, I25.2
Congestive Heart Failure	I09.9, I11.0, I13.0, I13.2, I25.5, I42.0, I42.5-I42.9, I43, I50, P29.0
Moderate/Severe Liver Disease	I85.0, I85.9, I86.4, I98.2, K70.4, K71.1, K72.1, K72.9, K76.5, K76.6, K76.7
Saddle Pulmonary Embolus	I26.02, I26.92
